# SEX DIFFERENCES IN MITOCHONDRIAL RESILIENCE: EVIDENCE FROM BABOON HEPATOCYTES

**DOI:** 10.1093/geroni/igac059.2928

**Published:** 2022-12-20

**Authors:** Daniel Adekunbi, Cun Li, Peter Nathanielsz, Adam Salmon

**Affiliations:** University of Wyoming, Laramie, Wyoming, United States; University of Wyoming, Laramie, Wyoming, United States; University of Wyoming, Laramie, Wyoming, United States; The University of Texas Health Science Center at San Antonio, San Antonio, Texas, United States

## Abstract

Events that occur in utero set the trajectory for later-life diseases and longevity. Compelling data exist for interactions between developmental programming and aging, but the underlying mechanisms are not clearly defined. Fetal exposure to glucocorticoids (GC) is associated with alteration in hepatic enzymes and metabolic function in later life. We previously reported increased hepatic lipid accumulation and obese phenotype in middle-age male baboons exposed to GC as fetuses. The mitochondria play significant roles in cellular processes including stress responses and possibly a nexus between developmental programming and aging. The present study investigated the long-term effects of in utero GC exposure on mitochondrial bioenergetics using hepatocytes derived from aging baboons (16–18 years, average lifespan 21 years). Mitochondrial bioenergetics of both left and right lobe liver hepatocytes were examined as well as potential sex differences in mitochondrial function. Cell viability following isolation was similar among sexes and liver lobes but hepatocytes from males were highly energetic compared to females. Significant bioenergetic differences were observed in hepatocytes isolated from female baboons’ left and right liver lobes, with higher basal, maximal, and ATP-linked respiration in left lobe hepatocytes compared to the right lobe. These lobe-specific bioenergetic differences were absent in males. Interestingly, H2O2-induced oxidative stress significantly modified male baboon hepatocyte bioenergetics but females were unaffected, suggesting mitochondrial resilience in females compared to males. These data demonstrate that early life exposure to GC elicits a sex-specific effect on mitochondrial function. These mitochondrial differences might drive differences in cell senescence between males and females.

